# Students’ Views on Vaccination against COVID-19 Virus and Trust in Media Information about the Vaccine: The Case of Serbia

**DOI:** 10.3390/vaccines9121430

**Published:** 2021-12-03

**Authors:** Iva Šiđanin, Biljana Ratković Njegovan, Bojana Sokolović

**Affiliations:** Department of Industrial Engineering and Engineering Management, Faculty of Technical Sciences, University of Novi Sad, 21000 Novi Sad, Serbia; iva.sidjanin@uns.ac.rs

**Keywords:** COVID-19 vaccinations, vaccines, students, media, Republic of Serbia

## Abstract

Mass immunization of the citizens of the Republic of Serbia began in January 2021. Information on the significance, manner, advantages and consequences of this process was intensively distributed through all communication channels, with the media playing a key role. According to the data of the official institutions for the public health of Serbia, by July 2021 the lowest percentage of vaccinated population was among those between the ages of 18 and 24—only 15% of this demographic had received the vaccine by this point. Given the low turnout of young people for vaccination, in this paper we investigated the general attitude of students in Serbia, as a special category of young people, towards the vaccine against the COVID-19 virus, as well as their attitude regarding information about vaccination in the media. Research was conducted on a sample of 345 students at the University of Novi Sad. The results of the research showed that 42% of students had not been vaccinated and did not plan to do so, 37.4% had received at least one dose of vaccine and 20.6% had not been vaccinated even though they planned to do so. Students who were vaccinated had more confidence in information provided through media channels than those who were not vaccinated. Therefore, it can be concluded that encouraging students to decide in favor of vaccination against the COVID-19 virus should come from the universities where they study as well as the media.

## 1. Introduction

The last two years have been marked on the global level by the appearance of a novel coronavirus, which has spread rapidly to many countries around the world and caused an epidemic, with a large number of infected and deceased people. The global implications of this epidemic have been negatively manifested in a number of fields, primarily in the medical, economic, social and political fields, and especially in the field of state readiness to manage crises of this type. 

As stated by Altunisik Toplu et. al., the new coronavirus was not identified in humans until 2019, although the first cases of pneumonia of an unknown etiology were observed in China (in the city of Wuhan, Hubei province). As early as 31 December 2019, the Chinese office of the World Health Organization (WHO) reported the appearance of this virus and identified it as the COVID-19 virus, whose characteristics are high infectivity and rapid spread among people around the planet [1]. This type of coronavirus causes severe respiratory distress syndrome, which dominates the clinical picture in infected individuals, and mortality from this disease is thought to be between 2% and 5% in the general population [2]. However, Sahin et al. state that elderly infected patients or patients with “underlying diseases (e.g., hypertension, diabetes, and chronic obstructive pulmonary disease) are considered high-risk, and their mortality rates are significantly higher and amount to >50%” [2] (p. 2988) according to [3]. In addition to this, the WHO stated that the new coronavirus is associated with the same family of viruses that cause severe acute respiratory syndrome—SARS—as well as with viruses that cause some types of colds [4].

At the end of 2020, the first doses of vaccines against the COVID-19 virus were on the world market. Serbia was the third country in Europe to start vaccination (24 December 2020), while Great Britain and Switzerland were the first two [5]. Less than a month later, on 19 January 2021, mass immunization of citizens with vaccines from one of four manufacturers began: Pfizer-BioNTech; Sinopharm; Sputnik V; and Oxford/AstraZeneca [6]. From that moment until the middle of September 2021, about 2.7 million people in Serbia were vaccinated—43.64% of the population [7,8], with about 6 million doses of vaccines [9]. The results of the research of the Alternative Report on the Position and Needs of Youth in the Republic of Serbia showed that 16.7% of the group of young people aged 20 to 24 were vaccinated in that period, as were 20.7% of young people aged 25 to 30 [10]. According to the official data of the Institute of Public Health of Serbia, published at the end of July 2021 [7], the lowest percentage of vaccinated people was seen amongst those between the ages of 18 and 24—only 15%—which soon proved to be a major social problem in stopping the pandemic.

The majority of students, a special category of young people, were hesitant to be vaccinated due to doubts related to the safety and efficacy of vaccines, given the speed and technology of their production [11,12].

This resulted in the fact that a person under the age of 25 was identified as a super carrier of the COVID-19 virus: educated, unvaccinated and does not respect the measures of physical distancing and wearing a protective mask [13].

In this regard, Barello et al. indicate that students’ attitudes towards vaccination differ from other age categories, primarily because they are “better educated, more open-minded, and respond more quickly to public health issues” [14] (p. 1470).

The leaders in the number of vaccinated students in 15 European countries, for which data were published [13], were Sweden (70%), Italy (60%) and Denmark (57%), while Serbia was in 12th place (20%). Behind it were the following Balkan countries: Montenegro (15%), Bulgaria (10%) and Bosnia and Herzegovina (1.5%).

The intention to receive vaccination among the population is generally influenced by a large number of factors. These are: socio-demographic characteristics; individual beliefs and experiences [15]; trust in the health system [16]; safety of the vaccine in terms of its composition; side effects, that is, harmful consequences; production speed; insufficient testing [17,18,19,20,21,22]; insufficient information; information provided through the Internet and social networking sites [23,24,25]; as well as high exposure to negative information about vaccination from the media (especially social media) according to [25]. It is worth noting that the WHO has declared the concept of vaccine hesitancy to be one of the top 10 threats to global health [26].

Recent research has shown that social networking sites have become a means of spreading misinformation about the COVID-19 virus [27], as they are a source of information for most young people (students), as well as those who are less likely to receive the vaccine [28]. Even the WHO, since the COVID-19 virus pandemic began, introduced the term “infodemia” [29] to denote the phenomenon of a large volume of fake news, misinformation and conspiracy theories appearing in public. On the other hand, Margolis et al. [28], indicate that traditional media are key channels of communication related to immunization. This corresponds to the findings of previous research which show that direct, non-sensationalist information from verified sources, as well as information placed through local media, increase the degree of acceptance of the vaccine [30].

Given these problems, the aim of this paper was to examine the attitude of students in Serbia towards vaccination, as well as the degree of trust in information about vaccination in the mainstream media. The results of the research showed that 42% of students have not been vaccinated and do not plan to do so, 37.4% have received at least one dose of vaccine, and 20.6% have not been vaccinated but plan to do so. Students who were vaccinated had more confidence in information placed through the media than those who were not vaccinated.

The significance of this paper is to examine the reasons for the delay in vaccination against the COVID-19 virus, especially in young academics. Serbia is one of the countries that first approved the vaccine and started mass immunization, but this slowed down abruptly. Therefore, the reasons for indecision and perception of vaccination risk in the student population were investigated. Particularly important are the data on the level of students’ trust in media announcements about the pandemic and the vaccine, which showed that, in addition to accepting the arguments about the need for vaccination, we also encountered opposite reactions. Furthermore, the importance of this research is reflected in the fact that it deals with a problem that transcends local and national borders; thus, comparing the data of our research with similar research conducted in other countries could contribute to creating a global strategy for educating and encouraging young people to be vaccinated against the COVID-19 virus.

## 2. Materials and Methods

Data were collected online from 6 to 26 July 2021, using an electronic questionnaire created via the Google Forms platform. The survey was reviewed and approved by the Committee for Ethical Issues of the University of Novi Sad (number: 01-82/67-1). The questionnaire contained a total of 22 questions, grouped into three parts: Socio-demographic questions; General attitude towards vaccination against the COVID-19 virus; Attitude towards information about vaccination placed through the media.

The questionnaire was distributed via email, Facebook and Viber, according to the principle of the snowball technique. The survey was anonymous and voluntary, while the questionnaire limited that only one response could be sent from each email address. A total of 345 students from the University of Novi Sad participated in the research. This university is, after the University of Belgrade, the second largest in terms of educational profiles and number of students and is one of the largest scientific and educational centers in Central Europe [31].

The processed data were described using appropriate descriptive statistics and analyzed using adequate nonparametric statistical methods and binary regression models. Numerical variables were summarized with mean, median, standard deviation, minimum and maximum, while categorical variables were summarized using frequencies and percentages. The normality of the variables was tested using the Kolmogorov–Smirnov test. Spearman rank correlation was used to measure the degree of association between two variables. Mann–Whitney U test and Kruskal–Wallis test were used to compare the medians of two or more independent groups. The crosstab procedure and χ2 test were used to examine differences in characteristics between groups for categorical variables. 

A binary logistic regression model was built to determine factors influencing students’ decision to vaccinate. The performance of the model was tested via Omnibus Tests of Model Coefficients, while the Hosmer–Lemeshow Test was used to measure the goodness of fit of the model. Moreover, for evaluation of the goodness of fit of the logistic regression model, two Pseudo R^2^ coefficients were calculated: Cox and Snell’s and Nagelkerke’s Pseudo R^2^. Both measures refer to the power of explanation of the model and represent the improvement of the model over the null model with no predictors. Regression results are presented via coefficients (B), standard errors (S.E.), Wald statistics, Significance level (Sig.), Odds ratios (Exp(B)), and 95% coefficients for odds ratios (C.I. for Exp(B)).

The level of significance in all tests was 0.05. 

All data were analyzed by using SPSS Statistics for Windows, Version 26.0 (IBM SPSS Statistics for Windows, Version 26.0. Armonk, NY, USA: IBM Corp).

## 3. Results

A total of 345 students answered the questionnaire. The average age of the respondents was 23 years. A total of 253 (73.3%) were female respondents, and most of them lived in urban areas. Most research participants were in undergraduate academic studies. Other demographic data are presented in Table 1.

### 3.1. Students’ Attitude towards COVID-19 Vaccination

Just over a third of the surveyed students had been infected by COVID-19. 31.1% had not had this virus, while 25.8% were not aware of whether, at some point, they had contracted the virus or not. Regarding vaccination, 58% stated that they had received or planned to receive a vaccine against the COVID-19 virus (20.6% plan, 37.4% vaccinated), while 42% had not and did not plan to be vaccinated (Table 2). 

Among unvaccinated students, there were 8.8% of those who declared themselves as anti-vaxxers.

The largest proportion (more than half—58.9%) of students who were vaccinated received the Pfizer-BioNTech vaccine, while 29.5% received the Sinopharm (BBIBP-CorV) vaccine, 7.8% received AstraZeneca COVID-19 (ChAdOx1 nCoV-19) vaccine and only 3.9% received Sputnik V (Gam-COVID-Vac), the smallest proportion. When asked what most influenced the choice of vaccine that they received, students’ main answer was: “recommendation of people I trust” (34.9%). In second place they cited preparation method (17.1%), while information through the media and rate of antibody production were the cited answers in 7% of cases, each. Doctor’s recommendation was the least cited answer (3.9%). 

As for reasons for not getting vaccinated, students most often said they were waiting for some more time to be aware of the potential consequences (28.0%). In second place, they stated that they did not have confidence in the quality of the vaccine (23.4%). Next was fear of side effects (13.7%), lack of belief in the effectiveness of the vaccine (10.3%) and that they still had antibodies (10%). Further statements included that they were afraid of difficulties in conceiving a child (7.3%), that they were against vaccination in general (4.6%) and that none of their friends had been vaccinated yet, so they did not want to either (2.7%).

**Figure 1:**
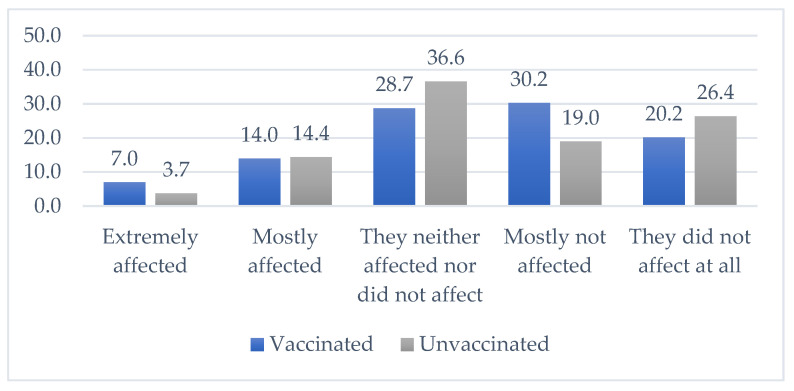
Influence of the media on decision to vaccinate/not vaccinate.

Both groups, vaccinated and unvaccinated, were asked to what extent the information put through the media influenced their decision to vaccinate or not vaccinate. Statistically significant difference was found between the decision to vaccinate and influence of information from the media (chi-square (4) = 10.499, *p* = 0.033). Results are shown in Figure 1 below.

To determine the factors influencing students’ decision to vaccinate, several tests using individual potential predictors were conducted and afterwards binary logistic regression model was built. The dependent variable that characterized students’ decision to vaccinate was coded in the following manner: a value of 1 was used if students had already been vaccinated (both or one dose) or planned to get vaccinated; otherwise, a value of 0 was used to represent the students who had not been vaccinated and did not plan to do it.

The considered independent factors were split into two groups. The first group comprised socio-demographic variables: gender; age; place of residence; education of parents; and if they had already suffered from COVID-19. The other group of variables consisted of the variables representing confidence in information providers: the media; health profession; social media; government; and public figures. 

Based on the data, there was no statistically significant relationship between gender and decision to vaccinate (chi-square (1) = 0.169, *p* = 0.681). However, there was a statistically significant difference in the age in these two groups of students (Mann–Whitney U = 12,426.5, *p* = 0.029). The students planning or already having been vaccinated were older in comparison to those that did not plan to get vaccinated (Table 3).

**Figure 2:**
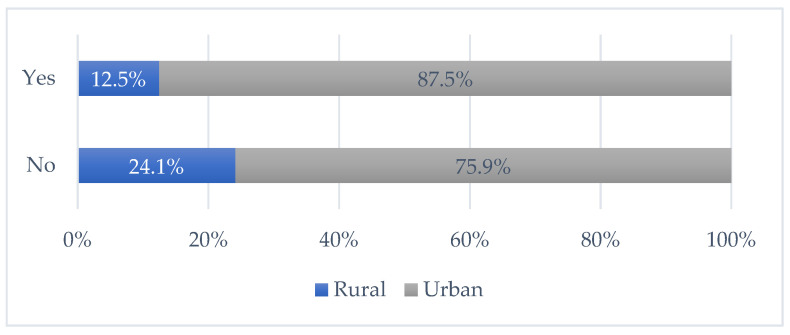
Place of residence and decision to vaccinate.

Another variable that had a statistically significant influence on vaccination was place of residence (chi-square (1) = 7.925, *p* = 0.005). A higher percentage of students from rural places of residences had a negative attitude towards vaccination when compared to the group of students from urban places (Figure 2). 

Both education of mother (chi-square (3) = 3.758, *p* = 0.153) and education of father (chi-square (2) = 4.408, *p* = 0.110) did not have a statistically significant influence on the decision to get vaccinated when considered individually. Furthermore, the fact that a student had suffered from COVID-19 also did not affect the decision to get vaccinated (chi-square (2) = 2.312, *p* = 0.315).

Next, we analyzed how confidence in media, health profession, social media, public figures and government had affected the students’ decision to get vaccinated. According to the results, there was a statistically significant difference in the level of confidence in the health profession (Mann–Whitney U = 7141.5, *p* = 0.000), in the media (Mann–Whitney U = 10,346.0, *p* = 0.000) and in government (Mann–Whitney U = 12,448, *p* = 0.01) between these two groups of students. On the other hand, there was no difference in the level of confidence in social media (Mann–Whitney U = 14,162.5, *p* = 0.69) and public figures (Mann–Whitney U = 14,357, *p* = 0.86). As can be seen in Table 4, students who were vaccinated or planned to do so had a statistically significant higher level of confidence in the health profession, in media and in government than those students who did not want to be vaccinated.

Now we present the logistic regression results and use all of the abovementioned predictors together to assess the prediction of the students’ decision to get vaccinated (Table 5). According to our Omnibus Tests of Model Coefficients, a model that included all predictors against a constant-only model was statistically significant (chi-square (11) = 107.079, *p* = 0.000). This result means that the set of chosen predictors reliably made distinction between students who had a positive and those who had a negative attitude towards getting vaccinated. Next, according to Hosmer–Lemeshow Test, the built model was adequate (chi-square (8) = 13.470, *p* = 0.097) and the model with all the predictors significantly better predicted the students who will get vaccinated in comparison to the model that had only intercept. Results showed that explanation of the model was average (Cox and Snell R square = 36%). The overall correct classification rate was 75%.

According to the results, the significant variables were: age; place of residence; mother’s education; and confidence in the media and the health profession. Older students were more likely to get vaccinated. Students from urban residences were also more likely to decide to take the vaccine. The higher the mother’s education, the more likely the student was to be vaccinated. Also, the higher the confidence in the media and the health profession, the higher the odds of getting vaccinated. In the model with all variables, confidence in government was no longer statistically significant.

### 3.2. Media Coverage of COVID-19 Vaccination

The largest percentage of students (42.3%) stated that they were partially informed about current topics, 14.2% were fully informed, 37.4% neither informed nor uninformed, while there was 3.2% uniformed and 0.9% completely uniformed.

Students were most informed about vaccines against the COVID-19 virus through the Internet (51.7%), followed by television (18.0%), social networking sites and different foreign media (12.5% each), radio (2.8%) and printed media (2.6%). In this sense, we investigated the level of students’ trust in the information provided by the mass media about vaccination against the COVID-19 virus.

On a scale of 1 to 5, students rated their degree of trust in the information received through the media on vaccination against the COVID-19 virus. Using Mann–Whitney U test, it was examined whether there was a difference in trust in the information in the media on vaccination among students who had been vaccinated and students who had not been vaccinated. The results showed that vaccinated and unvaccinated students significantly differed on this issue (Mann–Whitney U = 8040.5, *p* = 0.001). Students who were vaccinated had more trust (Md = 3.0) in information in the media than students who were not vaccinated (Md = 2.0).

The results of the research showed that, in relation to media announcements, the possibility of vaccination without strict scheduling (47.54%), as well as the opening of vaccination spots in public places (41.45%), bring greater stimulation for vaccination. In contrast, media coverage of vaccination—influencers (56.52%), government representatives (55.94%), the president (54.2%) and public figures (53.62%)—were assessed as demotivating activities. Also, the findings showed that 37.39% of respondents considered monetary incentives as a demotivating measure, while 29.28% of them believed financial incentives encourage vaccination. The remaining third of respondents did not have any attitude towards this measure. 

We also asked students which form of media they trusted the most when it came to informing the public about the vaccination process, and the most common answers were foreign internet portals (25%), foreign TV channels (15.4%), television channels with a national frequency (15%), domestic internet portals (9.7%), social networking sites (8.1%), daily newspapers (3.2%), local radio stations (1.8%) and radio stations with a national frequency (1.4%), while 5.7% of students did not trust any media at all.

**Figure 3:**
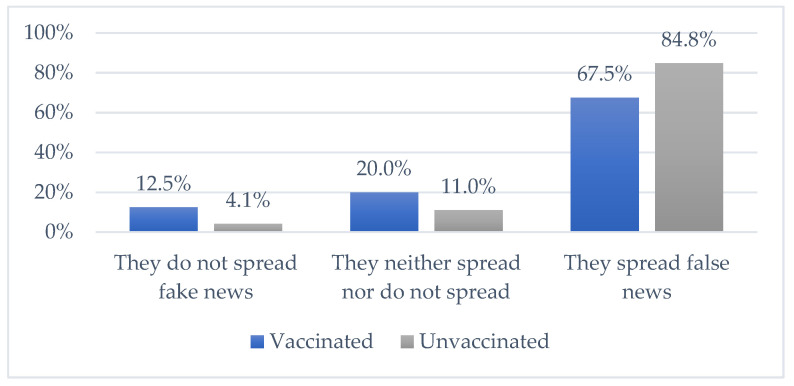
Is the media spreading false news regarding the vaccination against the COVID-19 virus?

The results of the research showed that the participants believed that much false news about vaccines and post-vaccination effects are placed through the media. Figure 3 shows the responses of vaccinated and unvaccinated students to this question. A statistically significant difference was found (chi-square (2) = 14.079, *p* = 0.001). A higher percentage of unvaccinated students thought that the media spreads fake news.

Next, students were asked through which media channels was the most misinformation spread, and television channels with a national frequency were mentioned in first place with 34.5% (in Serbia there are five television channels with national frequency, one public media service and four commercial channels). In addition, they also highlighted social networking sites (16.3%), daily newspapers (14.3%), domestic internet portals (13.6%) and, in a slightly smaller percentage, radio stations with a national frequency (3.9%), local radio stations (3%), foreign internet portals (1.8%), foreign television channels (1.8%) and weekly newspapers (1%).

## 4. Discussion

A survey conducted among students in the Republic of Serbia on their attitudes towards vaccination against the COVID-19 virus, as well as trust in media reports on the subject, showed a strong suspicion in relation to both issues. Given that slightly less than half of the surveyed students expressed a completely negative attitude towards vaccination, it is realistic to expect that even with all the measures taken by the state, the pandemic cannot be stopped faster.

There is a slightly greater interest in student vaccination among students around the world compared to Serbia. Over 68% of postgraduate students at the University of Singapore [32], 76.3% of students from China [12], as many as 80% of Canadian public university students [11] and 81.6% of Italian university students [14] were ready to be vaccinated, as well as 89.4% of undergraduate students in India [33], while the Middle East is among the regions with the lowest vaccine acceptance rate in the world [34].

An earlier, qualitative study conducted by Sandler et al. [35] on knowledge of vaccines pointed out that parents have a great influence on their children when it comes to decision-making. An indicative finding of our research is that, according to the participants, the level of education of their parents did not significantly affect their decision on vaccination (with the aforementioned finding obtained by crossing the data—the higher the level of education of the mother, the higher the probability of vaccination). This suggests two outcomes: either that professional and scientific arguments about the need for vaccination were not rationalized in the consciousness and attitudes of the participants; or that the negative attitudes of parents regarding vaccination, regardless of their level of education, affected their children’s attitudes.

The passage of time as an indicator of certain harmful consequences (28%), distrust in the quality of the vaccine (23.4%), fear of side effects (13.7%) and distrust in the effectiveness of the vaccine (10.3%) were the main reasons for non-vaccination of Novi Sad students. The obtained results correspond to the findings of similar research which indicate that the speed of vaccine introduction, efficacy, side effects, side effects, safety and vaccine choice are the main reasons for variability among students around the world [11,12,32,36]. 

At the beginning of the vaccination, the Government of the Republic of Serbia introduced a financial incentive (around €25) for each person who receives the vaccine. The results of the conducted research showed that 37.39% of the respondents thought that such a measure would not contribute to a higher vaccination rate, while 29.28% of them thought that financial stimulation encouraged vaccination. Although monetary incentives to encourage vaccination have been introduced by many countries, these measures have also been challenged, especially among ethics experts. For example, Professor Julian Savulescu [37], a philosopher and ethicist, called this model a “payment for risk model” from medical, social and economic aspects. Some other ethicists, such as Neal and Jecker, [37] have questioned the effectiveness of financial incentives, generally arguing that offering money to someone to do something may provoke interpretations that it is risky and that this, to some extent, adds pressure.

When it comes to trust in media reports about the need for vaccination, the conducted research showed that students who had more trust in media information approached vaccination without hesitation. Although we cannot directly correlate the ratio of media messages to vaccination decisions, trust in the media was significantly lower among unvaccinated students. Measuring the effects of media messages is a very delicate and complex process, which depends not only on the research methodology, but also on the researcher’s point of view, which can sometimes be based on the premise of strong media message effects, or on limited media message effects (also it is worth recalling Clapper’s doctrine, according to which the media strengthen much more than change existing opinions) [38]. This is all the more true because our research has shown that slightly less than half of the research participants stated that they were only partially informed on this topic, and slightly less of them were almost uninformed.

A similar study on how to communicate about vaccination against the COVID-19 virus [39], conducted among young people in Serbia, showed that they, although sometimes diametrically opposed, want to debate the topic of immunization, but also require much clearer communication in connection with vaccines by state authorities and the media. At the same time, they suggested that the domestic media should filter information more critically and create a larger number of educational programs about the general importance of vaccines, as well as about lesser-known facts about the characteristics of all available COVID-19 vaccines on the market. Besides, in addition to information placed through the domestic media, their views on the need for vaccination were greatly influenced by foreign media and drug agencies, due to which most respondents would not receive certain types of vaccine.

Margolis, Brewer, Shah, Calo and Gilkey [28] consider traditional media to be crucial in the process of promoting citizen immunization. Regardless of how much a media is trusted, the acceptance of vaccination is linked to information in national and local television, as well as newspapers [21]. On the other hand, Jain et al. [33] in their research emphasize the importance of social media in the process of informing students, pointing to the fact that those who are informed in this way hesitate in the process of vaccination. Rivas et al. [40] conclude that traditional media during the pandemic was perceived as transmitters and reporters on the development of the pandemic, while social networking sites played an important role in the faster dissemination of information, sometimes inciting fear.

In addition to trusting media information, it was important to determine the attitude of students towards fake news and misinformation. Rzymski et al. [29] believe that the easy availability and wide distribution of the Internet, forums and social media provide a fertile ground for the uncontrolled spread of false news and misinformation. Cardo, Kraus and Kaifie point to a wide range of misinformation that poses a global problem—from “denial, downplaying, or conspiracy theories to false and unsubstantiated claims regarding the origin of the virus and the inefficacy of cures and protective measures” [41] (p. 2). Some authors [42] even characterized the then President of the United States, Donald Trump, as the biggest initiator of false news about the coronavirus because he promoted dangerous and ineffective methods in the process of treatment and protection. The results of previous research have shown that high exposure to negative information about vaccination in the media, and even only short news that discourages the vaccination process [43], was associated with a lower level of vaccine acceptance [44,45,46], while high risk perception was associated with a large exposure to pandemic information via social media [40]. The obtained results confirm the perception of students, mostly unvaccinated, that a large volume of misinformation and false news is propagated by the media in Serbia, as well as the fact that this is most often carried out via television channels with a national frequency.

In general, it turned out that the greater the trust in the media and the health profession, the greater the chances of agreeing to vaccination. This presupposes the great responsibility of the mass media, objectivity and accuracy in reporting and a narrative that does not encourage panic nor creates doubt among citizens. When it comes to the media in Serbia, there is a pronounced polarization between the so-called pro-government and so-called opposition media. Unfortunately, this was already shown in the first months of the COVID-19 pandemic, when the problem was more politicized in the media than it was treated medically. Although with some reservations, we can state that such polarized reporting has contributed to the polarization of attitudes towards this disease in general, as well as towards the decision on vaccination.

Partial limitations of this paper are related to the design of the study, which may have influenced the possibilities of generalizing research findings, as well as the concretization of the application of results in practice. In addition, some of the email addresses of students that authors received officially proved to be invalid, which probably affected the lower response of potential research participants.

However, these limitations do not diminish the validity of this research and the credibility of the obtained data, but indicate the need for further research, primarily in terms of possible changes in young people’s attitudes towards vaccination, as well as the reasons for these changes. Given that the problem investigated in this paper, which is the attitude towards vaccination against the COVID-19 virus, is still very current, the research should be continued as longitudinal. This is all the more important because they would be based on the same phenomenon, i.e., on attitudes towards vaccination against the COVID-19 virus and on examining whether there have been changes over time in the ways of perceiving this problem among young people.

Furthermore, it is recommended to continue research among unvaccinated students in order to determine possible changes in attitudes towards vaccination, real reasons for refusing vaccination, as well as (dis)trust in media content related to informing about the need for vaccination.

## 5. Conclusions

In conclusion, for many citizens, and especially young people, the mass media, along with interpersonal and idiosyncratic networks, represent one of the most important sources of information on medical problems. Thus, they can significantly influence the public perception of media-relevant topics in the field of medicine, health and health policy. However, more information does not mean an increase in knowledge but, on the contrary, it can lead to greater confusion or misinformation. The media has great potential to convey positive health messages, so in this sense the purpose of media advocacy in the practice of health communication is to promote health goals, through strategic pressure on health and political institutions, but also on public opinion, in order to improve, on the one hand, certain health policies, and, on the other, the behavior of citizens related to health. Mass media also have the resources to, using managed information and so-called scientific journalism, contribute to a better understanding of the problem of vaccination and the removal of doubts related to public health caused by the pandemic. In particular, media should focus more on vaccine acceptance than on the distrust in vaccination and the medical establishment advocating these measures. 

## Figures and Tables

**Table 1:** Demographic data.

Variable	Category	Frequency (n)	Percentage (%)
Total		345	100
Gender	Male	92	26.7
	Female	253	73.3
Type of settlement	Rural	60	17.4
	Urban	285	82.6
Education	Undergraduate academic studies	201	58.3
	Integrated academic studies	47	13.6
	Master studies	77	22.3
	Doctoral (PhD) studies	11	3.2
	Other	9	2.6
Mother’s education	Elementary/high school	8	2.3
	Higher education	190	55.1
	MA/PhD	48	13.9
Father’s education	Elementary/high school	6	1.7
	Higher education	196	56.8
	MA/PhD	41	11.9

**Table 2:** COVID-19 and Vaccination.

Variable	Category	Frequency (n)	Percentage (%)
Total		345	100
Have you contracted the COVID-19 virus?	Yes	121	35.1%
	No	135	39.1%
	Don’t know	89	25.8%
Total		345	100
Have you received the COVID-19 vaccine?	Yes	129	37.4%
	No, but I plan to get vaccinated as soon as possible	71	20.6%
	No and I do not plan to get vaccinated	145	42.0%

**Table 3:** Age and decision to vaccinate.

	Average Age	Std. Deviation
Yes	24.1450	4.75785
No	23.0972	2.69763

**Table 4:** Descriptive statistics for confidence in information providers.

Variable	Vaccine	Median ± IQR	Minimum	Maximum
Confidence in health profession	No	2.4 ± 1.2	1.0	5.0
	Yes	3.2 ± 1.0	1.0	5.0
Confidence in media	No	1.5 ± 1.5	1.0	4.0
	Yes	2.0 ± 1.5	1.0	5.0
Confidence in social media	No	2.0 ± 2.0	1.0	5.0
	Yes	2.0 ± 2.0	1.0	5.0
Confidence in government	No	1.0 ± 1.0	1.0	5.0
	Yes	1.0 ± 1.0	1.0	5.0
Confidence in public figures	No	1.0 ± 1.0	1.0	4.0
	Yes	1.0 ± 1.0	1.0	5.0

**Table 5:** Logistic regression results.

Variable	B	S.E.	Wald	Sig.	Exp (B)	95% C.I. for EXP(B)	
						Lower	Upper
Gender	−0.40	0.30	1.72	0.19	0.67	0.37	1.22
Age	0.07	0.04	2.82	0.09	1.07	0.99	1.16
Residence	0.70	0.35	4.08	0.04	2.02	1.02	3.99
COVID-19 infected	0.11	0.17	0.43	0.51	1.12	0.80	1.55
Mother’s education	0.38	0.22	3.07	0.08	1.47	0.96	2.25
Father’s education	−0.07	0.22	0.10	0.76	0.93	0.60	1.45
Confidence in media	0.62	0.16	14.94	0.00	1.87	1.36	2.56
Confidence in health profession	0.99	0.19	27.72	0.00	2.69	1.86	3.89
Confidence in public figures	−0.19	0.18	1.10	0.29	0.82	0.57	1.18
Confidence in social media	−0.01	0.14	0.00	0.96	0.99	0.76	1.30
Confidence in politics	−0.12	0.19	0.39	0.53	0.89	0.62	1.28
Constant	−6.38	1.52	17.55	0.00	0.00		

## Data Availability

The data presented in this study are available on request from the corresponding author.
